# Metabolic and Clinical Consequences of Hyperthyroidism on Bone Density

**DOI:** 10.1155/2013/638727

**Published:** 2013-07-22

**Authors:** Jagoda Gorka, Regina M. Taylor-Gjevre, Terra Arnason

**Affiliations:** ^1^Department of Medicine, University of Saskatchewan, Saskatoon, Canada S7N 0W8; ^2^Division of Rheumatology, Department of Medicine, University of Saskatchewan, Saskatoon, Canada S7N 0W8; ^3^Division of Endocrinology and Metabolism, Department of Medicine, University of Saskatchewan, Saskatoon, Canada S7N 0W8

## Abstract

In 1891, Von Recklinghausen first established the association between the development of osteoporosis in the presence of overt hyperthyroidism. Subsequent reports have demonstrated that BMD loss is common in frank hyperthyroidism, and, to a lesser extent, in subclinical presentations. With the introduction of antithyroid medication in the 1940s to control biochemical hyperthyroidism, the accompanying bone disease became less clinically apparent as hyperthyroidism was more successfully treated medically. Consequently, the impact of the above normal thyroid hormones in the pathogenesis of osteoporosis may be presently underrecognized due to the widespread effective treatments. This review aims to present the current knowledge of the consequences of hyperthyroidism on bone metabolism. The vast number of recent papers touching on this topic highlights the recognized impact of this common medical condition on bone health. Our focus in this review was to search for answers to the following questions. What is the mechanisms of action of thyroid hormones on bone metabolism? What are the clinical consequences of hyperthyroidism on BMD and fracture risk? What differences are there between men and women with thyroid disease and how does menopause change the clinical outcomes? Lastly, we report how different treatments for hyperthyroidism benefit thyroid hormone-induced osteoporosis.

## 1. Introduction

The World Health Organization (WHO) defines osteoporosis as bone mineral density (BMD) 2.5 or more standard deviations below that of a young adult (*T* score) at any site. Characteristics of osteoporosis include increased risk of fragility fractures, a deterioration of bone architecture, and in addition to low bone mass. The distinction of secondary osteoporosis is applied to low BMD as a result of factors beyond postmenopausal and senile osteoporosis. The myriad of secondary causes includes glucocorticoid therapy, connective tissue disorders, malabsorption syndromes, diabetes mellitus, and hyperthyroidism [1]. This review will focus on the molecular and clinical consequences of hyperthyroidism on bone metabolism through a series of focused questions.

*Key Points regarding the Impact of Hyperthyroidism on Bone Health*

1. In hyperthyroidism, bone turnover is accelerated two-fold, with a net loss of bone.
2. Receptors for thyroid hormones and TSH are present in bone. These hormones may act directly on both osteoclasts and osteoblasts.
3. The severity of hyperthyroidism correlates with the decrease in BMD and the increase in fracture risk.
4. Hyperthyroidism affects cortical bone to a greater extent than trabecular bone and is best measured by BMD at the distal forearm.
5. Women over 65 years old with a TSH <0.1 have the greatest fracture risk.
6. Normalizing thyroid function alone is able to effect some reversal of bone loss.
7. Antithyroid therapy combined with Vitamin D and bisphosphonates is most effective at normalizing bone density. There is no role for calcitonin.
8. Subclinical hyperthyroidism is surprisingly prevalent, estimated to be present in up to 24% of those over age 60 who receive thyroxine replacement.
9. Subclinical hyperthyroidism contributes to an estimated additional 1% bone loss per year in these individuals.

## 2. Normal and Pathological Mechanism of Thyroid Hormone Action on Bone Metabolism

**Question 1**

How do the elevated thyroid hormone levels in thyrotoxicosis result in accelerated bone loss?

### 2.1. Normal Molecular Action of Thyroid Hormones on Bone Metabolism

As a result of pituitary TSH stimulation, the thyroid gland secretes two thyroid hormones, T4 (3,5,3′,5′-L-tetraiodothyronine) along with a small amount of T3 (3,5,3′-L-triiodothyronine), which is significantly more biologically active than T4. Additionally, T3 is peripherally created by the enzymatic conversion of T4 into T3 by the deiodinase enzymes D1 and D2. A third deiodinase enzyme, D3, converts T3 and T4 into inactive hormone metabolites reverse T4 (rT2) and rT3 [2]. Intracellular levels of T3 are determined by the relative action of the three deiodinases [3].

T3 action is mediated by its nuclear T3 hormone receptors (TRs). Two receptor isoforms, TR*α* and TR*β*, exist [4, 5] and their subtypes TR*α*1, TR*α*2, and TR*β*1 mediate T3 action though their wide distribution in tissues. There is extensive expression of thyroid hormone receptors within bone, indicating that thyroid hormones can influence this tissue type. Expression of TR*α* and TR*β* has been identified in chondrocytes, osteoblasts, and osteoblastoma and osteoclastoma cell lines [4, 6]. TR*α*1 expression in bone is about 10-fold higher than TR*β*1 [7], and studies of genetically modified mice have implicated TR*α*1 as the primary mediator of T3 action on bone [7–10]. The engineered absence of all TR*α*1 receptors in a mutant mouse model (TR*α* (0/0), display developmentally delayed ossification, growth retardation, and impaired bone mineralization despite being euthyroid, highlighting the requirement for thyroid hormone in normal bone metabolism. In contrast, TR*β* mutant mice TR*β* (−/−) exhibit normal skeletal development in young mice. There was a persistent elevation of T3 and T4 levels in these TR*β* (−/−) mice related to disruption of normal negative hormonal feedback at the hypothalamus and pituitary through TR*β*. The excessive stimulation of TR*α*1 in these adult mice from the elevated T3 and T4 levels resulted in accelerated bone remodeling leading to adult osteoporosis [7–11]. Clinically, this is similar to the observations in humans that prolonged hyperthyroidism results in accelerated bone loss.

Classically, the deleterious effects on BMD in hyperthyroidism have been attributed to high levels of the circulating thyroid hormones T4 and T3. However, more recent publications have also implicated low TSH levels alone in the development of reduced skeletal integrity. This is clinically relevant, as subclinical hyperthyroidism is defined by normal T3 and T4 levels with an isolated suppression of TSH and has been correlated with changes to human bone density over time [12–14]. The TSH receptor (TSHR) is predominantly expressed in cells of thyroid follicles; however, TSHR expression has also been demonstrated in a variety of tissue including osteoblasts and osteoclasts [15, 16], as well as kidney, brain, heart, testis, lymphocytes, and adipose tissue [17]. The presence of TSHR in osteoclasts and osteoblasts has led investigators to determine what direct effects TSH may exert on bone metabolism. Abe et al. [15] carried out experiments with mice carrying deletions for both (homozygous) or one (heterozygous) alleles of the TSHR. Homozygous TSHR deletion mice had low levels of T3 and T4 with high TSH, low BMD, and required exogenous thyroid administration for normal growth. Interestingly, the loss of BMD was not reversed with thyroid hormone supplementation, indicating that TSH acting through its hormone receptor is necessary for alterations in bone metabolism. Heterozygous mice had normal levels of T3, T4, and TSH; however, they also exhibited a significant reduction of BMD. Both groups of mice had osteoporosis due to high bone turnover, leading them to postulate that TSH action via its receptor acts as a negative regulator of bone turnover [15, 18]. Other studies have suggested that the action of TSH may also indirectly affect bone integrity through increasing deiodinase D2 activity in osteoblasts which results in local T3 elevations (by conversion of T4 to T3) as a consequence [19]. It remains unclear whether it is the action of excessive thyroid hormones, the lack of TSH, or a combination of both that is responsible for bone loss in the hyperthyroid state.

### 2.2. The Significance of Thyroid Hormone Excess on Rates of Bone Remodeling

The continuous process of bone remodeling through balanced resorption and deposition is essential to maintain the integrity and strength of the human skeleton [20]. During remodeling, osteoclasts degrade and resorb the old bony matrix, while new bone is deposited by osteoblasts. The normal bone remodeling cycle lasts 150–200 days (approximately <7 months), and any disturbances in this equilibrium lead to excessive/disordered bone formation or uncompensated bone loss. In the hyperthyroid state, the cycle occurs in roughly half of the normal time (3-4 months), and this accelerated rate of bone turnover creates an increased number of osteoclast resorption sites and increases the ratio of resorption to bone formation ultimately causing osteoporosis from cumulative new bone loss [21–23]. Using iliac crest biopsy specimens from hyperthyroid patients (*n* = 15) and age and sex-matched controls to reconstruct bone remodeling curves, it was determined that there was a 9.6% loss of mineralized bone with each cycle in the hyperthyroid group [24]. Conversely, hypothyroidism prolongs the remodeling process, resulting in reduced bone turnover and a 17% increase in mineralized bone as a consequence of a 700-day cycle [25].

### 2.3. The Significance of Thyroid Hormone Excess on Mineral Metabolism

The accelerated bone remodeling cycle in hyperthyroid states results in increased bone resorption and a subsequent increased release of calcium into systemic circulation [26, 27]. High levels of calcium, specifically in the ionized form, are present in up to 8% of patients with hyperthyroidism [26, 28]. High levels of serum calcium inhibit parathyroid hormone (PTH) secretion and create a negative calcium balance through prolonged urinary and fecal losses. Decreased PTH secretion causes hypercalciuria as a protective mechanism against hypercalcemia [28]. With low PTH, Vitamin D is not converted into its active form, leading to low gastrointestinal calcium and phosphorous absorption and resultant fecal calcium losses [29–31].

In addition to the release of calcium from bone into serum, levels of bone turnover markers are also elevated. Serum concentrations of alkaline phosphate, osteocalcin, osteoprotegerin, and FGF-23 correlate directly with the severity of biochemical hyperthyroidism [32–37]. Interestingly, bone turnover markers remain high for months after treatment due to the persistent increase in osteoblast activity, despite normalization of thyroid hormone levels in the serum [32–37].

In contrast, the urinary bone turnover marker, collagen-derived pyridinium cross-links, rapidly normalizes with correction of thyroid hormone serum levels. In the presence of elevated free T3 (fT3), collagen-derived pyridinium cross-links are elevated in urine; however, they rapidly return to normal shortly after initiation of therapy [38, 39].

## 3. Clinical Consequences of Hyperthyroidism on Bone Mineral Density and Fracture Risk

**Question 2**

Does the severity and duration of thyrotoxicosis correlate with the rate, extent and location of bone loss?

### 3.1. The Impact of Thyrotoxicosis on Bone Density (BMD)

Osteoporosis is a uniform feature of untreated and sustained thyrotoxicosis. Numerous reports have described a consistent decrease in BMD and increase in fracture risk in untreated overt hyperthyroidism [40]. This effect on BMD is evident in both pre- and postmenopausal women [41]. Hyperthyroidism differentially influences trabecular and cortical bone metabolism, with the latter more predominantly affected [42]. An early study demonstrated up to 40% increase in osteoclast resorption in cortical bone, compared to 2.7% reduction in trabecular bone volume [43]. Recently, a cross-sectional, population-based study of euthyroid and hyperthyroid women over 40 years of age evaluated the correlation between serum TSH levels and BMD of the distal and ultradistal forearm [44]. This is the site most profoundly affected by parathyroid hormone dysregulation [45]. The study, which reported a statistically higher risk of osteoporosis at the radius in hyperthyroidism, found a stronger association between low TSH and low BMD in distal (cortical) bone in comparison to the ultradistal (trabecular) bone of the radius, complementing previous publications recommending distal forearm BMD measurements as appropriate for studying thyroid effects on bone [42, 44]. Other areas of the skeleton that have demonstrated significant BMD decreases associated with hyperthyroidism include the lumbar spine, total hip and femoral neck. A 2010 cross-sectional study evaluating BMD in premenopausal women, as analyzed by DXA, revealed significantly lower BMD in hyperthyroid women compared to controls at the lumbar spine (0.928 versus 0.991), the total hip (0.838 versus 0.917), and the femoral neck (0.774 versus 0.832) [46]. In contrast to the distal radius, the decrease of BMD in the lumbar spine, total hip, or femoral neck does not appear to be associated with the duration of overt hyperthyroidism [46, 47].

The severity of hyperthyroidism appears to influence the degree of bone loss and increase the probability of osteoporosis when looking at the relative degree of TSH suppression, rather than looking at free T4 and free T3 levels. The aforementioned cross-sectional study by Svare et al. [44] reported a statistically significant decrease in BMD in hyperthyroid women with TSH <0.50 mU/L (reference range: 0.50–1.49 mU/L), with the highest association seen with TSH <0.10 mU/L. No statistically significant difference was observed in the BMD of hyperthyroid patients versus controls with TSH above 0.50 mU/L [44]. A 2007 analysis using data from the U.S. National Health and Nutrition Examination Survey (NHANES) evaluated the BMD in postmenopausal women and reported an odds ratio (OR) of 3.4 relating osteoporosis to TSH levels <1.8 mU/L, compared to an OR of 2.2 for TSH levels ≥1.8–4.5 mU/L [48]. The report also found that as TSH levels increased over the reference range (TSH 0.39–4.6 mU/L), a statistically significant increase was observed in BMD [48]. 

### 3.2. The Impact of Thyrotoxicosis on Fracture Risk

Thyrotoxicosis is an established risk factor for fracture later in life [49–51]. A prospective cohort study followed women over 65 years old for 4 years to evaluate fracture incidence and risk (*n* = 686) [52]. A TSH level of <0.1 mU/L resulted in a 4.5-fold risk of vertebral fracture and a 3.6-fold increase of hip fracture [52]. The fracture risk was much less pronounced with relatively higher TSH levels of 0.1–0.5 mU/L (normal range: 0.5–5.5 mU/L). The same report also found that a history of hyperthyroidism (regardless of duration or degree), after adjustment for TSH concentration and BMD, remains an independent risk factor for hip fracture [52]. Support for these finding was reported in a more recent cross-sectional study (*n* = 6722), which estimated a 31% higher fracture risk (forearm, hip, and vertebral) in patients with TSH <0.10 mU/L compared to TSH 1.0–1.49 mU/L [44].

## 4. Reversibility of Hyperthyroidism-Induced Bone Loss with Therapy

### 4.1. Targeted Antithyroid Therapy and Clinical Fracture Risk Outcomes

The choice of initial antithyroid therapy used to correct the hyperthyroidism may be clinically important, as highlighted by studies looking at subsequent fracture risk. In a historical follow-up study of 617 patients with toxic goiter, patients treated with radioactive iodine alone, rather than being cotreated with antithyroid medications, had an increased fracture risk at the spine and forearm in comparison to age- and gender-matched control [53]. This increase in fracture risk from controls was not observed in patients who were also cotreated with methimazole [53]. It was hypothesized by these authors that patients treated with combination therapy may have had more severe initial presentation of hyperthyroidism which resulted in earlier diagnosis and management, with subsequently less time for bone loss to occur [53]. It will be of great importance for future clinical management decisions to repeat this study in a larger controlled prospective manner to determine if duel initial therapy does in fact provide improved long-term protection from fractures.

**Question 3**

Is the bone loss or osteoporosis due to hyperthyroidism reversible by normalizing thyroid function?

The initiation of antithyroid therapy and successful achievement of an euthyroid state can reverse the extent of osteoporosis induced by overt hyperthyroidism [40, 54–58]. Numerous studies have evaluated BMD after the successful treatment of hyperthyroidism and reported a significant, though incomplete, recovery of bone density with effective antithyroid treatment within the first years after initiation of therapy [54, 56, 59–66]. Despite significant increases in BMD at the lumbar spine, femoral neck, and distal radius after 9–12 months of antithyroid therapy, bone density remained 5–16% lower than controls at 18–24 months of followup [50, 67].

At least three reports suggest after several years of euthyroidism, thyrotoxicosis-induced osteoporosis can be restored to normal [55, 56, 68]. BMD has been observed to return to a normal level, or that compared to healthy controls, after 3 to 6 years of euthyroid state after successful antithyroid therapy [40, 55, 65, 69].

### 4.2. Medical Treatment Options for Secondary Osteoporosis due to Hyperthyroidism

#### 4.2.1. Combination of Bisphosphonate and Antithyroid Therapy

Studies carried out with rodents have established that bisphosphonate therapy ameliorates the excessive bone loss caused by hyperthyroidism due to excessive T4 hormone supplementation [70]. Research in humans has evaluated the BMD and fracture risk outcomes using antithyroid drug monotherapy compared to the combination of bisphosphonates and antithyroid treatments. Lupoli et al. [71] examined the addition of alendronate to methimazole therapy in 40 pre- and postmenopausal hyperthyroid women and age-matched controls with no history of thyroid disease. BMD and osteocalcin levels were evaluated at baseline and 6 and 12 months after initiation of either methimazole (MMI) or MMI and alendronate combination therapy. At its conclusion, the study revealed a statistically significant clinical and molecular benefit in the combination therapy arm: a significant increase in BMD and decrease in the bone turnover marker osteocalcin. In addition this was independent of estrogen levels as both pre- and postmenopausal women exhibited significant increases in BMD and decreases in osteocalcin levels from basal values after 12 months of treatment [71]. Fittipaldi et al. [72] examined the effect of combination therapy in elderly male subjects with hyperthyroidism and osteoporosis. The results were similar to the effect seen in women; a detectable mean increase in BMD at the lumbar spine and femoral neck was significantly higher in patients treated with the combination of MMI and alendronate (6.2% and 2.1%, resp.) than in those treated with MMI alone (2.0% and 1.4%, resp.) [72]. Recently, another bisphosphonate, risedronate, were evaluated for its combination therapy effects on BMD and bone resorption markers in males with Graves' disease (*n* = 27, mean age = 43.7). Comparable with previous investigations, the increase in BMD at the lumbar spine, femoral neck, and distal radius was significantly higher after 12 months of combination treatment [73].

#### 4.2.2. Vitamin D

Therapy of hyperthyroidism-induced osteoporosis includes adequate Vitamin D supplementation and bisphosphonate therapy, in addition to antithyroid treatment. The continuous bone resorption and high serum calcium levels seen in sustained hyperthyroidism result in decreased levels of active Vitamin D, suggesting a therapeutic role for oral Vitamin D supplementation. A 2010 study examined the relationship between Vitamin D levels and BMD in patients with newly diagnosed hyperthyroidism [74]. The report revealed significantly lower mean *Z*-scores (age-matched controls) and overall BMD values in vitamin D deficient patients (<25 nmol/L) compared to hyperthyroid patients with sufficient vitamin D levels (>25 nmol/L) [74], supporting the inclusion of Vitamin D supplementation where deficiencies are detected.

#### 4.2.3. Calcitonin

As a result of its potent inhibition of osteoclast activity, calcitonin administration has also been investigated as a potential adjunct to therapy of hyperthyroidism-induced osteoporosis when used in combination with antithyroid medications. No additional benefit was seen in BMD or bone resorption markers at any dose of intranasal calcitonin although the BMD was significantly increased in all patients treated with antithyroid therapy and intranasal calcitonin at varying doses for 9 months [54]. The conclusion was that no additional benefit beyond reaching the euthyroid state was conferred by the addition of calcitonin [54].

## 5. Clinical Bone Disease in Subclinical Hyperthyroidism

**Question 4**

What impact does menopause or gender have on clinical outcomes of bone turnover in subclinical hyperthyroidism?

Subclinical hyperthyroidism is a biochemical definition, with suppressed or undetectable TSH levels and normal concentrations of T3 and T4. Subclinical hyperthyroidism incidence increases with age, especially in women, and is present in an estimated 1.5% of women over the age of 60 [75]. Serum TSH levels may spontaneously return to normal, as can be seen in patients with subclinical hyperthyroidism caused by Graves' disease or may progress to overt hyperthyroidism, more commonly in patients with autonomous thyroid nodules or multinodular goiters [75]. Exogenous hyperthyroidism is considered to exist due to iatrogenic overreplacement with thyroid hormone supplementation, such as during long-term management of differentiated thyroid carcinoma patients.

### 5.1. The Significance of Endogenous Subclinical Hyperthyroidism on BMD

Evaluation of the association between the presence of subclinical hyperthyroidism and the risk of development of osteoporosis has produced conflicting results from many small studies; therefore, BMD and fracture risk in this population are better examined by a subdivision dependent on estrogen levels found in pre- and postmenopausal women.

#### 5.1.1. Postmenopausal Women, BMD, and Subclinical Hyperthyroidism

Subclinical hyperthyroidism has consistently been correlated with an increased risk of reduced BMD [12, 13, 50, 76–78]. Foldes et al. [14] conducted a cross-sectional study (*n* = 37) measuring BMD in pre- and postmenopausal women with endogenous subclinical hyperthyroidism. It was observed that the BMD was not significantly affected in premenopausal patients, in direct contrast to postmenopausal women who had significantly decreased BMD at the femoral neck and radius, sites of predominantly cortical bone.

#### 5.1.2. Premenopausal Women, BMD, and Subclinical Hyperthyroidism

Multiple studies of premenopausal women with subclinical hyperthyroidism, either in comparison to normal controls or postmenopausal subjects, have consistently found normal or near-normal BMD levels. It is unclear if such studies have been able to remove the bias of age, as postmenopausal women may just have had longer durations of low TSH [13, 14, 76, 78–84]. Two small studies have reported statistically significant reduction in BMD in the femoral neck, but not in the lumbar spine, of premenopausal women, in comparison to significant reduction at both sites in postmenopausal patients [84, 85]. Similarly, the majority of studies have revealed normal serum markers of bone turnover, with the exception of the aforementioned studies, which noted significant increase in bone resorption markers in both pre- and postmenopausal women [14, 80, 82, 86, 87].

#### 5.1.3. Males, BMD, and Subclinical Hyperthyroidism

Nearly half of the cases of osteoporosis in men are found to have an underlying secondary cause. The effects on BMD in male patients with endogenous subclinical hyperthyroidism have not been widely studied or reported. A recent retrospective analysis of hip fracture risk in patients with subclinical thyroid dysfunction revealed a hazard ratio (HR) of 4.91 in men with endogenous subclinical hyperthyroidism in comparison to a HR of 2.42 in postmenopausal females of the same category [88]. The reason for the increased fracture risk in males is unexpected, and further statistical analysis of androgens, TSH, T4, and T3 levels may help clarify the risks involved.

### 5.2. The Importance of Endogenous Subclinical Hyperthyroidism on Increased Fracture Risk

Subclinical hyperthyroidism has also been implicated with an increased incidence of fracture at any site. A retrospective study (*n* = 2004) examined fracture risk patients with subclinical hyperthyroidism, defined as a TSH below the reference range (≤0.4 mU/L) and a normal total T4 and T3, to age- and gender-matched controls [89]. The results revealed a hazard ratio of 1.25 for osteoporotic fracture. This association was lost, however, when patients were included who converted into either overt hyperthyroidism or the euthyroid state during the follow-up period (median: 5.6 years) [89].

## 6. Reversibility of Subclinical Hyperthyroidism-Induced Bone Loss with Therapy

**Question 5**

How well does low bone density recover through correction of subclinical hyperthyroidism alone?

There is an established link between subclinical hypothyroidism and BMD decreases in postmenopausal women, leading investigators to test the efficacy of antithyroid therapy on bone density recovery in these patients. Studies in this subset of patients with subclinical hyperthyroidism have revealed a strong association with improvement in BMD within as little as 6 months of reaching euthyroidism [64, 90–92]. A prospective study by Mudde et al. [76] followed postmenopausal women with subclinical hyperthyroidism for a 2-year period following treatment with MMI and compared them to untreated controls. Although significant changes in bone turnover serum markers were not seen in either group, the mean BMD in women treated with MMI was significantly higher than in those in the untreated group [76]. Faber et al. [93] conducted a prospective study examining the effects of radioiodine therapy on the BMD of postmenopausal women with subclinical hyperthyroidism secondary to nodular goiter. The spine BMD in the treated patients increased by 1.9% at 1-year followup and remained increased by 1.5% after 2 years. BMD at the hip was also increased by 2.3% after 1 year of treatment and remained 1.7% increased after 2 years. These results were significant when compared to the untreated controls, in whom BMD decreased by about 2% per year at the hip and the spine [93]. A recent prospective study of seventeen women over the age of 65 with subclinical hyperthyroidism due to nodular goiter and treated with radioiodine showed a similar effect on BMD after achievement of euthyroidism [94]. After 1 year of treatment with radioiodine, average BMD had increased by 1.9% at the femoral neck and by 1.6% at the lumbar spine in the twelve patients who achieved euthyroidism. Conversely, in the four patients who continued to have subclinical hyperthyroidism despite one year of therapy, the average BMD had decreased by 2% at the femoral neck and by 1.8% at the lumber spine at followup [94].

As mentioned previously, subclinical hyperthyroidism in premenopausal women is typically associated with a normal BMD and no alteration in serum bone turnover markers. Therefore, the usefulness of treatment of this group has been put into question. Nonetheless, a small prospective randomized trial evaluated BMD in premenopausal women after 6 months of antithyroid therapy and concluded that no difference in BMD between treated patients and controls was found [95]. This is an important finding for clinical management, as no measureable clinical benefit to bone health through active treatment was detected.

## 7. Iatrogenic Subclinical Hyperthyroidism and Negative Outcomes to Bone Density

**Question 6**

How common is subclinical hyperthyroidism in the general population and what clinical consequences does this have on their bone health?

### 7.1. Prevalence of Iatrogenic Subclinical Hyperthyroidism

The prevalence of exogenous subclinical hyperthyroidism is much more common than the endogenous form, due to the widespread use of thyroid hormone supplementation and its inclusion in many herbal and weight loss formulations [75]. An estimated 3% of women over the age of 60 are taking exogenous T4 (thyroxine) for medically indicated purposes, either for TSH suppression or as replacement of iatrogenic or endogenous hypothyroidism [96]. Full replacement doses of levothyroxine are crudely approximated at <1.6 *μ*g/kg, and suppressive doses result in a decreased TSH serum level [97]. In a community-based study of 1210 adults over 60 years old, Parle et al. [96] evaluated the prevalence of thyroid dysfunction. It was found that 24% of these patients were overreplaced with thyroid hormone, having measurably low TSH levels. Congruent with this report, a recent review of subclinical thyroid disease by Cooper and Biondi [75] estimated low TSH levels in 20–40% of patients treated with thyroid hormone. These studies highlight the surprising prevalence of community subclinical hyperthyroidism.

### 7.2. The Impact of Exogenous Subclinical Hyperthyroidism on Bone Mineral Density

Euthyroid patients who are treated with oral T4 and sustain serum TSH levels within the normal reference range have not been found to have changes in their BMD regardless of menstrual status [6, 98]. Because of wide variations in outcomes between pre- and postmenopausal women, the extent of bone loss associated with exogenous thyroid therapy is best examined individually within these groups of women.

#### 7.2.1. Postmenopausal Women

As with endogenous subclinical hyperthyroidism, postmenopausal women taking excess exogenous thyroid hormone have been found to be at risk for hyperthyroid-induced skeletal effects [99]. Significant reductions of BMD in postmenopausal women treated with thyroxine are a universal finding [34, 61, 83, 100–104]. A meta-analysis by Faber and Galloe [90] reported a significant reduction of BMD by 9% after 10 years of thyroxine treatment in postmenopausal women compared to controls. This value implies that subclinical hyperthyroidism induced by thyroxine administration confers an additional annual bone loss of nearly 1% in postmenopausal women who already have an estimated 1–2.5% annual bone loss. Uzzan et al. [105] demonstrated similar loss values in their meta-analysis, with BMD losses of 7% of the spine, 5% of the femoral neck, 9% of the trochanter and Ward's triangle, and 7% of the distal radius. These values may correspond to a 12–44% lifetime risk of hip fracture conferred from bone loss of 6–10% over a 10-year period [106]. Clearly, these studies highlight the need for clinical vigilance in optimizing replacement levels to reduce adverse effects on bone metabolism.

#### 7.2.2. Premenopausal Women

The main reason for minimal bone loss in premenopausal women is thought to be due to preserved estrogen production; therefore, the impact of thyroxine overreplacement on bone mass should exhibit a lesser impact than postmenopausal women [97]. Consistent with this notion, suppressive doses of thyroxine have not been implicated in significant reductions of BMD in premenopausal women in large population analyses and review [6, 90, 99, 105, 107, 108]. A meta-analysis revealed that BMD in premenopausal women treated with thyroxine for 8.5 years (90% with TSH below the reference range) had a mean reduction in BMD of 2.7%, a value corresponding to 0.3% annual bone loss throughout the duration of treatment, a number found to be nonsignificant by the investigators in comparison to controls [90].

#### 7.2.3. Males

Few studies have included male patients evaluating the effects of exogenous thyroid hormones on BMD. Meta analyses and literature reviews have concluded that no significant effect on BMD has been observed in men receiving suppressive thyroxine therapy [6, 99, 107]. 

### 7.3. Fracture Risk in Subclinical Hyperthyroidism due to Exogenous Overreplacement

There is an increased risk of bone fracture in individuals who are overreplaced with exogenous thyroid hormone. Fracture risk is related to the degree of TSH suppression (TSH < 0.1 mU/L versus TSH 0.1–0.5 mU/L) and patient factors, such as age [52]. For women with TSH values in the normal range, T4 replacement does not confer a risk for fracture [52]. A recent observational cohort study examined fracture risk in patients >18 years old (mean age: 60.3 females, 61.8 males) on long-term T4 therapy with a median follow-up of 4.5 years (*n* = 17, 684) [109]. No increase in fracture risk was found in patients with low TSH concentrations (0.04–0.4 mU/L) compared to normal range TSH levels (0.4–4.0 mU/L); however, they reported a two-fold increase in fracture risk in patients who had undetectable TSH levels (≤0.03 mU/L) [109]. Fracture risk is further increased in postmenopausal women with suppressed or undetectable TSH levels (<0.1 mU/L) due to T4 replacement with as much as a four-fold increase in vertebral and hip fractures after 4 years of followup compared to controls with normal TSH values (>0.5 mU/L) [52]. Therefore, the greatest risk lies with those with the lowest TSH values and those who are postmenopausal.

**Question 7**

Can bone loss due to exogenous thyroid hormone overreplacement be reversed?

#### 7.3.1. Efficacy of Correction of Thyroid Replacement Dosing on Bone Recovery

Appropriate thyroxine supplementation in euthyroid individuals who maintain TSH levels within the reference range has not been implicated in BMD reduction [6]. It has been suggested that titration of suppressive thyroxine therapy is imperative in protecting the negative skeletal effects of subclinical hyperthyroidism [97]. In a small clinical trial, Appetecchia [110] examined the effects on BMD and thyroxine suppression therapy in 200 pre- and postmenopausal women with nodular goiter. The result revealed that thyroxine therapy titrated to doses that normalized TSH levels (0.27–4.20 mU/mL) did not have any deleterious effects on BMD regardless of menopause status [110]. First-line management clearly indicates that thyroxine dosing should be monitored and adjusted to prevent ongoing bone loss.

#### 7.3.2. Calcium Supplementation

A prospective study by Kung and Yeung [83] evaluated the role of calcium supplementation in 46 postmenopausal women treated with suppressive doses of thyroxine. The women were divided into groups of either 1000 mg calcium daily or placebo and followed for 2 years. At 6-month intervals and at the conclusion of the study, BMD was determined for the study participants. The results revealed that the patients supplemented with calcium had stable BMD, whereas patients in the placebo cohort had significant bone loss of 5% at the lumbar spine, 6.7% at the hip, 4.7% at the trochanter, and 8.8% at Ward's triangle, and BMD was significantly lower than the supplemented group [83]. The researchers concluded that suppressive thyroxine therapy should be supplemented with calcium to prevent BMD loss in treated patients [83].

#### 7.3.3. Adjunct Estrogen Replacement Therapy in Postmenopausal Women

Effects of exogenous subclinical hyperthyroidism on bone are minimal in premenopausal women presumably due to the positive effects of estrogen on skeletal health. A cross-sectional study by Schneider et al. [97] evaluated the impact of concomitant estrogen therapy in 991 postmenopausal women taking levothyroxine in replacement or suppressive doses. Women treated with estrogen and thyroid hormone has significantly higher BMD than those taking thyroid hormones alone. BMD in the estrogen replacement group was 12.9% higher at the midshaft radius, 8.1% higher at the hip, 7.8% higher at the lumbar spine, and 17.7% higher at the ultradistal radius [97]. With adjustments for age, BMI, smoking, and concurrent use of corticosteroids and thiazide, the BMD in women taking both levothyroxine and estrogen was comparable to that of women solely taking estrogen, without use of thyroid hormone [97]. The positive effects of estrogen are presumed to oppose the negative calcium balance seen in the hyperthyroid state, by increased absorption and decreased excretion of calcium [111]. However, the direct effect of estrogen on osteoblasts and the increase in calcitonin following estrogen administration are also thought to play a role. Although estrogen is clearly effective in modulating bone metabolism and was routinely prescribed prior to the Women's Health Initiative, it is a controversial supplement. Given the efficacy of alternative, nonhormonal therapies (below) estrogen is not recommended solely for the prevention or treatment of osteoporosis [112].

#### 7.3.4. Bisphosphonates

Bisphosphonates are indicated for the treatment of osteoporosis induced by overt hyperthyroidism. In a randomized trial, Rosen et al. [81] evaluated the efficacy of bisphosphonates in patients taking suppressive doses of thyroxine who fit the biochemical criteria for subclinical hyperthyroidism (normal freeT4 in 97% of cases). Men and postmenopausal women were randomized to either taking thyroxine and placebo, or thyroxine with addition of 30 mg of intravenous pamidronate every 3 months and were followed for 2 years. The bisphosphonate-treated group showed significant increases in BMD at the spine (4.3%), total hip (1.4%), and trochanter (3.0%) compared to patients solely receiving thyroid hormone [81].

#### 7.3.5. Calcitonin

Similar to the conclusions discussed previously in overt hyperthyroidism, the use of intranasal calcitonin has not been found to benefit BMD in patients treated with exogenous subclinical hyperthyroidism. Kung and Yeung [83] found no additional benefit of intranasal calcitonin on BMD in postmenopausal women supplemented with 1000 mg of calcium daily.

## 8. Conclusions and Key Points

Regulation of skeletal development and maintenance of skeletal integrity is regulated in part by a normal balance of thyroid hormones. Rodent studies have implicated both thyroid hormones and pituitary TSH in regulation of bone remodeling, although it remains unclear if T3, TSH, or the combination of both are responsible for the increased turnover and subsequent osteoporosis observed in the hyperthyroid state. Overt hyperthyroidism leads to osteoporosis and increased fracture risk in patients regardless of their age or gender. Prevention of fractures attributed to low BMD in hyperthyroid patients begins with antithyroid therapy, as reversibility of hyperthyroid-induced osteoporosis is evident a few years after achievement of an euthyroid state. Supplementation with Vitamin D and bisphosphonates may further increase BMD in these patients. The fine control of bone integrity by thyroid hormones is exhibited by the detrimental consequences on BMD and increased fracture risk by subclinical hyperthyroidism in postmenopausal women and men, and in postmenopausal women treated with suppressive doses of thyroid hormone. Postmenopausal women and men with endogenous subclinical hyperthyroidism should be considered for bone density measurement and subsequent antithyroid therapy. Postmenopausal women on thyroid supplementation in suppressive doses should have bone density testing, with appropriate titration of suppressive therapy, and supplementation with calcium and bisphosphonates.
